# Universal and Specific Predictors of Chinese Children With Dyslexia – Exploring the Cognitive Deficits and Subtypes

**DOI:** 10.3389/fpsyg.2019.02904

**Published:** 2020-01-08

**Authors:** Shuang Song, Yuping Zhang, Hua Shu, Mengmeng Su, Catherine McBride

**Affiliations:** ^1^State Key Laboratory of Cognitive Neuroscience and Learning, Beijing Normal University, Beijing, China; ^2^College of Teacher Education, Capital Normal University, Beijing, China; ^3^Sichuan Research Center of Applied Psychology, Chengdu Medical College, Chengdu, China; ^4^State Key Laboratory of Cognitive Neuroscience and Learning & IDG/McGovern Institute for Brain Research, Beijing Normal University, Beijing, China; ^5^Elementary Education College, Capital Normal University, Beijing, China; ^6^Department of Psychology, The Chinese University of Hong Kong, Hong Kong, China

**Keywords:** dyslexia, phonology, morphology, subtypes, Chinese

## Abstract

While previous studies have shown that the impact of phonological awareness (PA) and rapid automatized naming (RAN) on dyslexia depends on orthographic complexity in alphabetic languages, it remains unclear whether this relationship generalizes to the more complex orthography of Chinese. We investigated the predictive power of PA, RAN, and morphological awareness (MA) in dyslexia diagnosis status in a sample of 241 typically developing and 223 dyslexic Chinese-speaking children. Compared with the control group, children with dyslexia performed notably worse on character reading and all three cognitive measures. A logistic regression analysis showed that PA and RAN were both significant predictors, while MA also played a relatively important role for predicting dyslexia status in Chinese children. In the next step, we used multigroup analyses to test if these three cognitive predictors were of the same importance in predicting reading variance in different reading proficiency groups. And the results showed that the regression coefficient of MP is stronger for the control group than the dyslexia group, while the regression coefficient of PD tends to be stronger for the dyslexic group. Further cluster analysis identified four subtypes of dyslexia in this sample: a global deficit group, a phonological deficit group, a RAN deficit group, and a mild morphological deficit group. Our findings are largely consistent with previous studies of predictors of dyslexia, while uniquely demonstrating the differences in predictive power of these three cognitive variables on reading, as well as the unique contribution of MA in Chinese reading.

## Introduction

Developmental dyslexia is a specific disorder characterized by dysfluent or inaccurate word recognition that is not attributable to sensory deficit, insufficient education, or low IQ (Ramus et al., 2003). As researchers now widely support a multiple deficits model of Chinese reading difficulties (Ho et al., 2002; Zhou et al., 2014; Peng et al., 2017), one of the leading questions researchers are focusing on is what are the important cognitive profiles of dyslexia in Chinese. In addition, given that there is no firm consensus from previous research (Ho et al., 2002; Liu et al., 2006; Wu et al., 2009), investigation of different subtypes of dyslexia is also a matter of interest. As it is relatively subjective to set a criteria for cognitive deficits in multiple-case analysis, cluster analyses based on a large sample can provide more reliable evidence about heterogeneous of dyslexia, as a data-driven method.

The multiple cognitive deficits model of reading difficulties proposed a multi-factorial etiology for this complex developmental disorder (Pennington, 2006). Accordingly, a number of cognitive causes have been put forward as of fundamental importance, ranging from cognitive anomalies possibly existing since long before formal education is received [deficits in phonological awareness (PA), or naming speed, for example] (Ziegler and Goswami, 2005; Furnes and Samuelsson, 2011), to deficits emerging with the acquisition of literacy and as consequences of impaired reading (such as orthographic deficit) (e.g., Ho et al., 2004; Xue et al., 2013). Therefore, in the present study, which aimed to examine the multi-deficit hypothesis in children from mainland China and to shed light on early markers for dyslexia, we only included assessments of the pre-literacy cognitive areas.

Research on predictors of reading abilities and disabilities has identified PA and rapid automatized naming (RAN, a measure of naming speed) as particularly strong indicators, both concurrently and longitudinally, even after statistically controlling for children’s IQ (Wolf and Bowers, 1999; Ramus et al., 2003; White et al., 2006; Smythe et al., 2008; Furnes and Samuelsson, 2011). In comparison with normal controls, significant impairments in PA, and RAN have commonly been observed in dyslexic children (Pennington, 2006; Landerl et al., 2013). Although phonological skills have been suggested as an especially reliable predictor in predicting reading variation, evidence has shown that the strength of the relationship between the phonological deficit and reading varies with orthographic depth (Frost et al., 1987; Ziegler et al., 2010; Moll et al., 2014). Landerl et al. (2013) tested 1,138 typically developing children and 1,114 children with dyslexia across six orthographies with varying levels of consistency, and found that PA and RAN are both strong predictors of dyslexia diagnosis status. More interestingly, they reported that the impact of both cognitive domains is greater in complex orthographies (e.g., English) than in less complex orthographies (e.g., Finnish). As only alphabetic languages were involved in Landerl’s study, it would be interesting to know whether the essence of how orthographic complexity influences reading can be generalized to Chinese, an even more complex orthography.

Over the past decade, research on predictors of dyslexia and poor reading in Chinese children has reported that both PA (McBride-Chang et al., 2011; Xue et al., 2013) and RAN (Ho et al., 2004; Pan et al., 2011) are strongly associated with children’s reading variations, and dyslexic children were also observed as having significant deficits in PA and/or RAN (Shu et al., 2006; Chung et al., 2011). However, some other studies reported inconsistent findings: PA is not a significant predictor of Chinese word reading among beginning readers after controlling for RAN, orthographic skills, and morphological awareness (MA) (Tong et al., 2009; Yeung et al., 2011). A recent meta-analysis of Chinese dyslexia found that the predictive power of PA for reading disability is not stable (Peng et al., 2017). The inconsistencies across these results may be partly due to the recruitment of relatively small samples of dyslexic children. Perhaps more importantly, there might be other significant factors correlated with learning to read Chinese, such as MA.

The morpheme, as the smallest meaningful unit, provides basic semantic information within a language. MA refers to the ability to manipulate morphemes and employ word information rules in one language (Shu et al., 2006). One of the most prominent characteristics of Chinese is that the language contains a large number of homophones, with Mandarin having an average of five homophones corresponding to each syllable, taking tones into consideration. For example, the syllable/qing1/can represent more than six characters with different meanings [e.g., 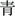
(blue), 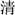
(clear), 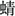
(dragonfly), 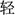
(relax), 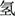
(hydrogen), 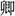
(a minister or a high official in ancient times)]. This phenomenon leads to ambiguity when only sound is used to distinguish words in Chinese. Thus, to become a successful reader one needs to be able to distinguish the meanings of words that sound identical (Shu et al., 2006; Tong et al., 2009). A series of Chinese studies have demonstrated that MA is associated with literacy skills and reading disability (McBride-Chang et al., 2003; Li et al., 2012; Liu et al., 2013). MA has been found to be both a concurrent and longitudinal predictor of reading in typically developing children (Lei et al., 2011; Liu et al., 2013). Moreover, MA has also been found to be one of the best factors that distinguishing dyslexia Chinese children from their age-matched controls (Shu et al., 2006; Zhou et al., 2014; Tong et al., 2017)., and children with severe reading deficits generally show more sever deficits in MA (Peng et al., 2017). Given the aforementioned property of Chinese, it is not hard to understand why MA has been thoroughly accepted as both a strong concurrent and a longitudinal predictor of Chinese literacy skills (Chen et al., 2009; Yeung et al., 2011; Pan et al., 2015). Therefore, in the present study, a morphological production task (suitable for older children) was also included to tap into children’s MA (e.g., Shu et al., 2006).

Another set of studies devoted to identifying subgroups of dyslexia has revealed that people with dyslexia who have a deficit in PA constitute the most commonly identified subgroup across languages (Ho et al., 2004; White et al., 2006), while some studies have found that children with dyslexia also have difficulty with rapid naming (Katzir et al., 2008; Jednoróg et al., 2014). Compared with multiple-case analysis, cluster analyses avoid debate over the criteria for cognitive deficits by adopting a data-driven method for the classification of subgroups (Heim et al., 2008; Jednoróg et al., 2014).

With different subtypes reported across studies, it is possible that the dyslexic population is heterogeneous, with varying degrees of impairment in different cognitive skills (White et al., 2006; Ho et al., 2007). Unlike alphabetic languages, Chinese is characterized by its morphosyllabic writing system where 90% of the Chinese characters consist of two components: the semantic radical gives a clue to meaning and the phonetic gives a clue to pronunciation. In addition, new words in the Chinese language are made up of novel combinations of existing syllables, and not through the coining of new syllables. Dyslexia subtypes in Chinese samples have therefore been found to show characteristics specific to the language itself (Ho et al., 2002; Shu et al., 2006). For example, Wu et al. (2009) report observing that, in a group of 75 Chinese children with dyslexia, the largest proportion (96%) exhibited deficits in MA, compared with 53% with deficits in PA and 45% with deficits in RAN. Until now, there has been no firm consensus on which subtypes of dyslexia exist among Chinese speakers, and this necessitates further exploration.

To summarize, the current study addressed one research question from two dimensions. First, with the same case-control design and data-driven logistic regression analyses as used as in Landerl et al. (2013) research, the current study aimed to test whether these preliteracy cognitive areas of PA, RAN, and MA are important predictors in a large sample of Chinese children (*n* = 464), and whether these cognitive variables are contributing differently in predicting reading between dyslexia and normal developing children. The second goal was to identify specific dyslexic subtypes in the Chinese language based on an investigation of the three above-mentioned cognitive domains.

## Materials and Methods

### Participants

Ethical approval for the present study was obtained from the Institutional Review Board at Beijing Normal University. In total, 223 individuals with dyslexia and 241 typically developing controls participated in the present study. All were children born in Beijing, China, and were native Mandarin speakers, with normal IQ and no reported mental, physical, or sensory difficulties.

Children diagnosed with dyslexia were recruited from eleven elementary schools in Beijing, attended by a total of about 3,600 children aged between 9 and 11 years. Children with dyslexia were identified and selected using the following procedure: (1) as recommended by their Chinese teachers, the lowest 20% school reading academic performance children (total *n* = 708) in each class were invited to participate the screening test for dyslexia; (2) Of these children, those who scored at least 1.5 SD below their respective grade mean on the Chinese character reading (CR) (Xue et al., 2013) were included. This threshold was based on previously used criteria (Shu et al., 2006); (3) Those with either performance IQ or full-scale IQ scores lower than 85 on the Wechsler Intelligence Scale for Children (C-WISC; Gong and Cai, 1993) were excluded. The remaining children were identified as having dyslexia, and thus participated in data collection (see section “Measures” below); and (4) On the basis of parental reports on a rating scale for attention and hyperactivity behavior, data from children identified as having attention deficit hyperactivity disorder (Swanson, Nolan, and Pelham –IV Teacher and Parent 18-Item Rating Scale, Swanson et al., 2001) were additionally excluded from analyses.

This resulted in a sample of 201 dyslexic children (mean age = 130.70 ± 17.07 months; 155 boys). Using the same criteria as described above, a further 22 children with dyslexia (mean age = 124.36 ± 4.29 months; 17 boys) and with a normal IQ score of ≥25th percentile on the Raven’s Standard Progressive Matrices were identified from an ongoing longitudinal cohort study (Lei et al., 2011), resulting in a total sample of 223 children with dyslexia.

The control group consisted of 241 children, all from the aforementioned longitudinal cohort (mean age = 125.27 ± 3.55 months; 128 boys). The inclusion criteria were: (1) a score no more than 1 SD below the grade mean on the same character reading test as mentioned above, and (2) a normal IQ score of ≥25th percentile on the Raven test. Subtests of C-WISC test were administered for only 131 children in control group.

### Measures

#### Chinese Character Reading

This task was used to measure children’s untimed reading accuracy (Lei et al., 2011). The CR task consists of a list of 150 single Chinese characters, which the children were asked to name; self-corrections and guessing were allowed. All of these characters are expected to have been learned by grade six in Beijing (Shu et al., 2003). The final score was the total number of characters that a child correctly named. Cronbach’s alpha for the CR task is 0.94.

#### Phoneme Deletion

The PD task was used to measure children’s PA (Pan et al., 2015). Participants were required to delete a target phoneme from a monosyllabic Chinese word (e.g., “Say/shu1/without the/sh/”). The target phoneme for each item was the first, middle, or final phoneme. The test consisted of two practice items and 26 experimental items, and the final score was the number of correctly answered experimental items. Cronbach’s alpha for the PD task is 0.83.

#### Rapid Automatized Naming of Digits

The RAN task consisted of a 5 × 10 matrix of digits that children were required to name as quickly and accurately as possible (Pan et al., 2011). This task was administered twice, and the mean total naming time across the two trials was taken as the final score. The test–retest reliability of the RAN task is 0.92.

#### Morphological Production

The MP task has been widely used in previous studies to measure Chinese children’s MA (Shu et al., 2006). During the test, participants were orally presented with 15 two-character compound words with one of the morphemes highlighted as the target (e.g., in the word 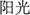/yang2guang1/, meaning sunshine, the target morpheme was 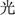/guang1/). They were then required to orally produce two new words containing the same Chinese character as the target morpheme. In one of these cases, the morpheme represented by this character in the new word should be the same as the target morpheme (e.g., a one-point answer in the above case was 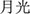/yue4guang1/, meaning moonlight). In the other case, the morpheme represented by this character should be different from the original target morpheme (e.g., 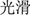/guang1hua2/, meaning smooth, contains a homograph morpheme with a different meaning and would be scored as one point). Answers not produced according to the guidelines were scored as zero. The final score was the number of correct words given during the task, with a maximum score of 30. Cronbach’s alpha for the MP task is 0.80.

### Statistical Analyses

Raw scores on all measures were converted to grade-specific *z*-scores according to a previous large-scale reading study (Xue et al., 2013); these *z*-scores were entered into all subsequent analyses. The grade-specific *z*-scores for the RAN digits test were multiplied by –1; thus, higher scores represented better performance, as for the other measures. Deficits in the cognitive domains of PA, MA, and RAN were defined as a grade-specific *z*-score below –1 SD.

#### Predictive Analysis

SPSS 20.0 was used to conduct a logistic regression analysis (Peng et al., 2002). The Hosmer–Lemeshow test was used to assess the goodness of fit of the model. Scores on each of the three cognitive skill tests, namely PD, RAN digits, and MA, were introduced as predictors of dyslexic status, in accordance with the following model:

$$P\,\left(\mathrm{diagnosis}\,\mathrm{with}\,\mathrm{dyslexia}\right)=1/\left\{1+\exp\left[-\left(\beta_{0}+\beta_{1}\times {\left(P\,D\right)}_{i}+\beta_{2}\times {\left(R\,A\,N\right)}_{i}+\beta_{3}\times {\left(M\,A\right)}_{i}+\varepsilon_{i}\right)\right]\right\}$$

#### Multi-Group Analysis

Linear regression analyses were used to examine the predictability of PD, RAN, and MA on children’s reading ability separately for dyslexics and normal controls. To test the equality across the control and dyslexic groups, multi-group analyses were conducted to test the possible different effects of these cognitive skills on the reading performance. The chi-square difference test comparing constrained models and freely estimated models was used to evaluate the model.

#### Subtype Analysis

In order to explore the subtypes within the dyslexic sample, cluster analysis (Rousseeuw, 1987) was used, as follows: (1) Hierarchical clustering was first used to determine the number of subgroups based on the three cognitive measures of PD, RAN, and MA, with larger changes in agglomeration coefficient representing a better-fitting number of clusters (Jurowski and Reich, 2000); (2) Subsequently, the *k*-means technique was applied to identify the final clusters. In the first step, between-groups linkage was used to combine dyslexic children into clusters during hierarchical clustering. Each squared Euclidean distance between two data points was calculated as a measure of similarity of two participants. The best cluster solution was selected by visual inspection of the agglomeration coefficients. The Average Silhouette Width was also used to suggest the “best number” of clusters. In the second step, k-means clustering was used to maximize cluster homogeneity and the number of clusters was decided from hierarchical procedure. this approach revealed the characteristics of different patterns of dyslexia. We then used ANOVA and Turkey’s *post hoc* test to test the differences among subgroups of dyslexia and control group separately for all three cognitive skills and the reading measures. This approach revealed the characteristics of different patterns of dyslexia.

## Results

Descriptive statistics for raw scores and grade-specific *z*-scores for reading and the three cognitive tests are presented in Table 1. Compared with control group children, children with dyslexia performed notably worse on the character reading task (CR: 0.33 vs. – 2.45). The additional clear group differences in scores on the three cognitive skills tasks suggested that these three profiles could be useful in distinguishing children with and without dyslexia. Correlations among reading and cognitive measures are shown in Supplementary Table S1, with all measures significantly intercorrelated.

**TABLE 1:** Descriptive statistics for all reading measures for control and dyslexia groups.

	**Raw mean (SD)**	***Z* Mean (SD)**	**Min**	**Max**	**Skewness**	**Kurtosis**
**Controls (*n* = 241, 53% boys)**						
PD	18.85 (1.66)	0.08 (0.92)	−3.74	0.73	−1.81	3.00
RAN	15.91 (3.55)	0.13 (0.92)	−4.42	2.16	−1.13	2.84
MP	22.97 (3.42)	0.18 (0.88)	−2.38	1.99	−0.41	−0.29
CR	118.48 (9.93)	0.33 (0.69)	−0.94	1.75	0.08	−0.89
**Dyslexics (*n* = 223, 77% boys)**						
PD	12.27 (5.19)	−1.70(1.54)	−5.29	2.47	−0.20	−0.31
RAN	20.82 (4.29)	−1.12(1.22)	−5.89	1.34	−0.96	1.45
MP	16.93 (4.05)	−1.47(1.09)	−4.73	0.99	−0.37	0.00
CR	85.14(13.39)	−2.45(1.03)	−8.88	−1.52	−2.42	8.58

*PD, Phoneme deletion; RAN, rapid automatized naming digits; MP, morphological production; CR, Chinese character reading.*

**FIGURE 1:**
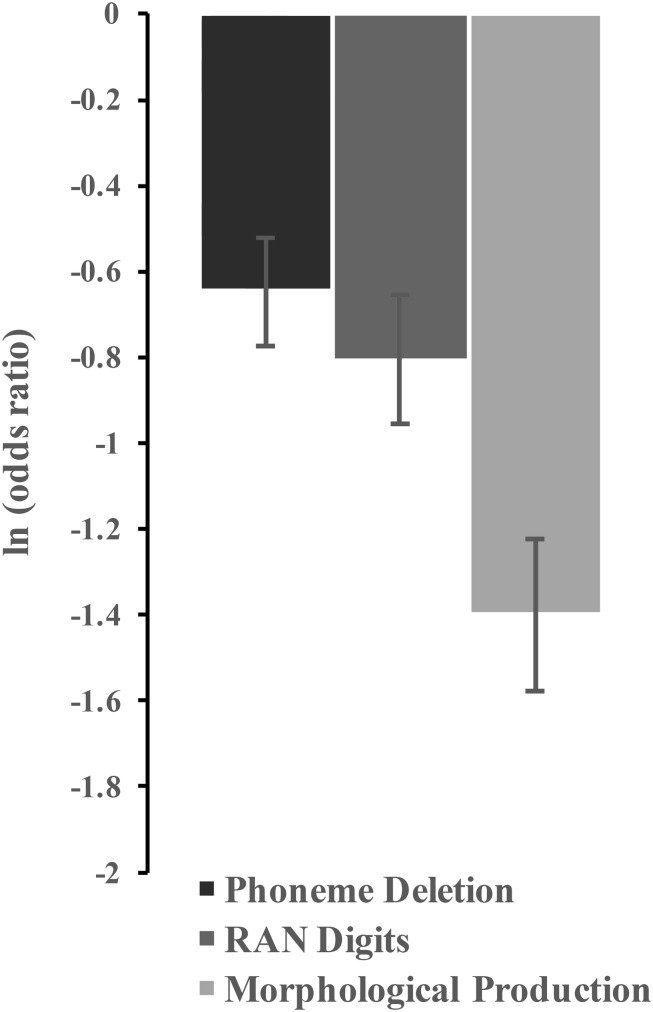
Estimate (ln OR) for Phoneme Deletion, RAN Digits and Morphological Production, respectively (vertical bars represent one standard error).

Next, PD, RAN digits, and MP test scores were introduced as predictive variables in a logistic regression model. The corresponding odds ratios (OR) and coefficient estimates (ln OR) derived from the Wald statistic are presented in Table 2 (Model 1). As expected, PD, RAN digits, and MP scores were all reliable predictors of dyslexia status. Participants with lower PD, RAN digits, or MP scores were at an increasing risk of having being diagnosed with dyslexia (PD: OR = 0.52, 95% CI [0.41, 0.67]; RAN digits: OR = 0.45, 95% CI [0.33, 0.60]; MP: OR = 0.25, 95% CI [0.17, 0.35]). As shown in Figure 1, the association was stronger in the case of MP than in the case of either PD or RAN digits. A logistic regression analysis controlling for gender, age, block design, and similarities was also conducted for children to whom the C-WISC test had been administered (*n* = 343), and similar predictive patterns were observed for the three core cognitive measures (Model 2 in Table 2).

**TABLE 2:** The logistic regression model for predicting dyslexia.

	**Estimate**	**S.E.**	**Odds Ratio (OR)**	***p-*Value**	**95% C.I. for OR**	
					**Present study**	**Landerl et al., 2013**
**Model 1^a^ (*n* = 464)**						
PD	−0.65	0.13	0.52	<0.001	[0.41, 0.67]	[0.31, 0.41]
RAN	−0.81	0.15	0.45	<0.001	[0.33, 0.60]	[0.31, 0.41]
MP	−1.40	0.18	0.25	<0.001	[0.17, 0.35]	–
Cox & Snell *R* Square					0.511	
**Model 2^b^ (*n* = 343): 212 dyslexics and 131 controls**						
Gender	0.83	0.45	2.30	0.065	[0.95, 5.58]	
Age	0.06	0.02	1.06	0.002	[1.02, 1.09]	
Block design^c^	−0.39	0.09	0.68	<0.001	[0.57, 0.81]	
Similarities^d^	−0.18	0.11	0.84	0.104	[0.67, 1.04]	
PD	−0.68	0.18	0.51	<0.001	[0.35, 0.72]	
RAN	−0.66	0.21	0.52	0.002	[0.34, 0.79]	
MP	−1.20	0.25	0.30	<0.001	[0.18, 0.49]	
Cox & Snell *R* Square					0.578	

*PD, Phoneme deletion; RAN, rapid automatized naming digits; MP, morphological production; CR, Chinese character reading. a, full sample included. b, only children with WISC-R scores included. c, d, in WISC-R.*

In the next step, we used regression analyses to test if these three cognitive predictors were of the same importance in predicting reading. As can be seen in Table 3, the results differed for the dyslexia and control groups. For the control children, both RAN (β = 0.23, *P* < 0.001) and MP (β = 0.31, *P* < 0.001) were strong predictors, while PD was not (β = 0.07, *P* = 0.249), indicating that better performance on RAN and/or MP was associated with better performance in reading. For the dyslexic children, PD (β = 0.25, *P* = 0.001) and RAN (β = 0.13, *P* = 0.048) were two strong predictors, while MP was not (β = 0.09, *P* = 0.206), indicating that poor PA and/or slower RAN, but especially PA, was usually combined with poor performance in reading. The change in the χ^2^ values when each predictor was constrained to be equal for these two groups during multi-group analyses was also included in Table 3. There was no significant change when the prediction strength between RAN and CR was constrained to be equal. However, the regression coefficient of MP is stronger (χ^2^ = 4.026, *p* = 0.045) for the control group than the dyslexic group. Moreover, the regression coefficient of PD tends to be stronger (χ^2^ = 3.246, *p* = 0.072) for the dyslexic group.

**TABLE 3:** Standardized coefficients in linear regression and multi-group analyses.

	**CR**					
	**Dyslexic group**		**Control group**		**Multi-group analyses**	
	**β**	***p***-**Value**	**β**	***p***-**Value**	***χ*^2^**	***p***-**Value**
PD	0.252	0.001	0.069	0.249	3.246	0.072
RAN	0.131	0.048	0.233	<0.001	0.806	0.369
MP	0.088	0.206	0.313	<0.001	4.026	0.045

*PD, Phoneme deletion; RAN, rapid automatized naming digits; MP, morphological production; CR, Chinese character reading.*

Subsequently, cluster analysis was carried out to explore subtypes in the dyslexic sample. Change in the agglomeration coefficients suggested that a two-cluster or four-cluster model would best capture the data. When referring to the average silhouette width, the value for two-cluster solution and which for four-cluster solution are both local maximum values. However, the two-cluster solution only showed an overall assessment of high or low performance and did not reveal the differences among subgroups. In order to understand the features of different deficit patterns more clearly, the four-cluster solution was applied. To validate the results of the four-group cluster analysis, discriminant analysis was used. As suggested by the leave-one-out classification during discriminant analysis, 96.4% of original grouped cases were correctly classified.

**FIGURE 2:**
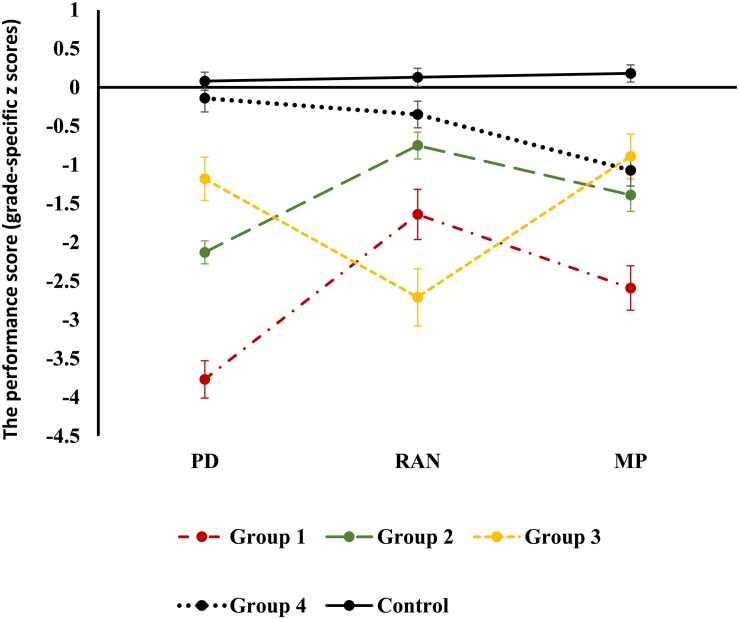
Comparison of cognitive scores in four deficit groups of dyslexic children against controls (in grade-specific *z* scores), with error bar representing 95% confidence interval.

Figure 2 presents the characteristics of the deficit patterns for the four groups, which were labeled as a global deficit group (Group 1), a phonological deficit group (Group 2), a RAN deficit group (Group 3), and a mild morphological deficit group (Group 4). Participants in the global deficit group exhibited difficulty in all three cognitive domains (Table 4), with grade-specific *z*-scores all below –1.5. Specifically, participants in this group performed particularly poorly on PD (*z* = –3.77) and MP (*z* = –2.59). The phonological deficit group obtained the lowest scores on PD (*z* = –2.13) compared to the performance of children in other groups, with the exception of the global deficit group. Children in the phonological deficit group also exhibited poor MA (*z* = –1.39), while their performance in RAN was only moderately impaired (*z* = –0.75), although still lower than that of the control group. Along with poor PA (*z* = –1.18), the RAN deficit group scored the lowest of all four subgroups on RAN (*z* = –2.71). However, their performance in MP was no worse than that of children in either the phonological deficit group or the mild morphological deficit group. Children in the mild morphological deficit group exhibited difficulty only in MP (*z* = –1.07), with almost normal performance on both the PD and RAN tasks.

**TABLE 4:** Means (SD) grade-specific Z-scores of the classification measures of control and four deficit groups.

		**Dyslexia subgroups**				
	**Control (241)**	**Group 1 (48)**	**Group 2 (69)**	**Group 3 (35)**	**Group 4 (71)**	***F* value**
PD	0.08 (0.92)_a_	−3.77 (0.85)_e_	−2.13 (0.63)_d_	−1.18 (0.84)_c_	−0.14 (0.76)_b_	266.83^∗∗∗^
RAN	0.13 (0.92)_a_	−1.64 (1.14)_d_	−0.75 (0.74)_c_	−2.71 (1.11)_e_	−0.35 (0.74)_b_	100.63^∗∗∗^
MP	0.18 (0.88)_a_	−2.59 (1.02)_c_	−1.39 (0.90)_b_	−0.89 (0.86)_b_	−1.07 (0.88)_b_	124.39^∗∗∗^
CR	0.33 (0.69)_a_	−2.97 (1.51)_c_	−2.55 (0.97)_c_	−2.48 (0.74)_c_	−1.97 (0.48)_b_	332.88^∗∗∗^

*PD, Phoneme deletion; RAN, rapid automatized naming digits; MP, morphological production; CR, Chinese character reading. Group 1, global deficit; Group 2, phonological deficit; Group 3, RAN deficit; Group 4, mild morphological deficit. ^∗∗∗^*p* < 0.001. Means in the same row that do not share subscripts differ at *p* < 0.05 on Turkey’s *post hoc* test.*

Participants in the four subgroups also showed heterogeneity in their CR task scores (Table 4). Children in the mild morphological deficit group only scored lower than those in the control group in CR (cohen’s *d* = –3.87, *p* < 0.001), and performed better than the other three subgroups (Group 1: cohen’s *d* = 0.89; Group 2: cohen’s *d* = 0.76; Group 3: cohen’s *d* = 0.82; *p*s < 0.001). With performance significantly worse than those in the control and mild morphological deficit groups, children in the other three dyslexia subgroups performed comparably on the Chinese character reading (Group 3 vs. Group 2: cohen’s *d* = 0.08; Group 2 vs. Group 1: cohen’s *d* = 0.33; Group 3 vs. Group 1: cohen’s *d* = 0.41; *p*s > 0.05).

## Discussion

With a relatively large sample of 223 dyslexic children and 241 typically developing children, the present study investigated to what extent various cognitive variables predicted children’s diagnostic status, and the differences in predictive power of these variables on reading. In addition, characteristics of different dyslexia subtypes in Chinese had also been examined. Similarly to the findings for alphabetic languages (Landerl et al., 2013; Moll et al., 2014), our results also showed that deficits in PA and RAN tests were both strong predictors of dyslexia status. However, a deficit in MA was the best predictor of Chinese dyslexia. As predicting individual variances in children’s reading, MA showed stronger predictability in the control group, while PA tends to be a better predictor in the dyslexia group, and RAN was of equal importance for both groups. Furthermore, we identified four subgroups of different deficit patterns and suggested that more severe dyslexics were particularly more impaired in these three cognitive domains. However, it is worth noting that all four subgroups exhibited moderate to severe deficits in MA and that the observation of heterogeneity in Chinese dyslexia is consistent with previous findings (Ho et al., 2004; Shu et al., 2006; Wu et al., 2009).

Both PA and RAN in the present study, either with the morphological factor included (Table 2) or excluded (Supplementary Table S2), significantly distinguished dyslexia status. What’s more interesting is that these cognitive skills differed in predicting individual differences for Chinese children with and without dyslexia. Compared with the control group, PA showed a stronger prediction on reading in the dyslexia group. One possible explanation could be that PA developmentally serves as a base for children learning to read (Hong et al., 2018), and it may contribute to reading acquisition through its impact on MA (Pan et al., 2015). As a result, phonology would be more influential for the lower-proficiency Chinese readers. For children at a higher level of reading achievement, variance in reading accuracy is reduced, and similar variance reductions can also be found in PA. Thus, the role of PA may become less salient with reading experience. Similarly, some previous studies showed for readers in five alphabetic languages that the predictive power of PA became weaker as mastering of orthography–phonology correspondence became easier (such as in highly transparent languages) (Ziegler et al., 2010; Furnes and Samuelsson, 2011). Together with the results from the present study, these findings underscore the universality of the importance of PA in reading, and shed light on the need to pay attention to different proficiencies for understanding the phonology–reading relationship.

In line with previous findings in alphabetic languages and Chinese (Kirby et al., 2010; Landerl et al., 2013; Zhou et al., 2014; Peng et al., 2017), RAN significantly predicted children’s diagnostic status and individual variances in reading. Furthermore, our multi-group analyses revealed that the predictability of RAN on reading is comparable between the dyslexic and control groups, suggesting a dominant role of this skill in Chinese children across different reading proficiencies. Establishing fluency in reading, involving automatic sequencing of Chinese characters, is of particular importance to become a skilled reader. The rapid number naming task tapped this ability across reading levels, such that those who were faster and more effective at the orthographic–phonology accessing in one domain (i.e., numbers), also tend to be better readers in the domain of character reading.

More importantly, our study suggested that MA, in addition to PA and RAN, appears to be an even more important cognitive construct in Chinese dyslexia. This was in line with a series of previous studies of Chinese reading development and impairment (McBride-Chang et al., 2003; Chen et al., 2009; Tong et al., 2017). It is interesting to note that MA exhibited greater predictive power than did phonological processing skills in the present study. This is partially due to the properties of the Chinese writing system and the nature of the process of learning to read Chinese. Categorized as a morphosyllabic language, Chinese is relatively semantically transparent; therefore, mastering the meaning of the character or morpheme is vitally important in learning to read, especially in new characters and word learning. Moreover, Chinese contains many homophones, so knowing the meaning of morphemes may greatly help children to distinguish these homophones and thus improve their reading ability (e.g., McBride-Chang et al., 2003; Shu et al., 2006). Thus, it is no wonder that MA was found to be more important for the skilled readers, the controls, in the present study. In Chinese, semantic information is reflected by the morphological properties of words. Therefore, MA is key for Chinese character recognition, a widely used measure in dyslexia diagnosis (Li et al., 2012), as it helps children to distinguish the meanings of different characters.

Four subgroups of children with dyslexia were identified in the present study and these subtype characteristics also supported our findings in the predictive analyses. When morphological skills were comparable (Groups 2 and 3 vs. Group 4), poor PA and/or slower naming speed would block one’s possibility of becoming a better reader, suggesting phonology as an essential factor in deficient readers. On the other hand, with poor PA and slower naming speed (Group 1 vs. Group 3), additional inferior skills in morphology only slightly impair children’s reading and indicate a diminished function of MA. Along with the aforementioned findings, it is clear that morphological skills exhibited a more important role in skilled readers as compared with deficient readers. This is because for a beginning reader, most of the characters to be learned are simple characters, which are more or less directly meaningful (Shu et al., 2003). Thus, correspondence among orthography, phonology, and meaning are relatively transparent. For a skilled reader, however, due to the characteristics of the Chinese language, the ability to distinguish between homophones and homographs becomes increasingly important. As a result, the importance of MA gradually emerges. To summarize briefly, as the importance of different cognitive skills varies, it is necessary to provide differentiated educational strategies for children of different reading proficiency.

In addition, children in the first three groups (the global deficit group, phonological deficit group, and RAN deficit group) all showed moderate to severe deficits in PA. Thus, phonological deficits represented one dominant characteristic across the whole sample (68.2%). This is similar to what has been found in previous multiple-case studies, in which phonological deficits have been found to emerge in the vast majority of participants with dyslexia (Ramus et al., 2003; White et al., 2006), but differs from the findings of Ho et al. (2002, 2004), who observe that only 15.3 to 29.3% of children with dyslexia in Hong Kong exhibit deficits in phonology. This disparity may be partially explained by the different forms of instruction used in the relevant education systems as well as differences between Mandarin and Cantonese. In Hong Kong, a whole-word and drilling approach is used, in which children must retrieve the pronunciation of characters by rote (Ho et al., 2004), while children in mainland China learn to assemble the sounds of characters through the pinyin phonetic system. The latter approach emphasizes the importance of phonological processing in reading.

There were some limitations to the present study. First, although we intended to investigate the predictability of preliterate cognitive skills, it might have been also interesting to have included some postliterate cognitive domains (orthographic awareness for example). Second, the present findings are all based on a cross-sectional design. With this study, in addition to several previous studies of Chinese reading impairment, we are beginning to get a better understand of the essential role of these cognitive skills. However, more longitudinal studies should be introduced to examine the contribution of these skills over time. Moreover, in future studies, reading impairments in other literacy domains should be considered, such as in reading fluency, spelling, and reading comprehension.

Despite these limitations, the current findings suggest some important conclusions about Chinese dyslexia. First, our findings highlight the important effects of MA, in addition to the effects of PA and the data from RAN tests, in understanding developmental dyslexia. The predictive power of morphological processing in explaining dyslexia in Chinese speakers suggests the necessity of acquiring knowledge about morphemes, which may help one to become a more skilled reader. Second, the contribution of the three cognitive skills differed across children’s reading proficiency, indicating that differential educational strategies should be taken into consideration in teaching (more attention should be addressed on phonological rules for beginning readers and/or slow learners, for example). Finally, our findings indicate that dyslexic Chinese children are heterogeneous, and the majority of children exhibited double or multiple deficits, which provided additional support for the multiple-deficit hypothesis for Chinese developmental dyslexia.

## Data Availability Statement

The datasets for this manuscript are not publicly available because: the present data belongs to an ongoing longitudinal study and most of the data are still in processing and need to be protected. Requests to access the datasets should be directed to HS, shuh@bnu.edu.cn.

## Ethics Statement

The studies involving human participants were reviewed and approved by the Institutional Review Board of Beijing Normal University Imaging Center for Brain Research and the State Key Laboratory of Cognitive Neuroscience and Learning. Written informed consent to participate in this study was provided by the participants’ legal guardian/next of kin.

## Author Contributions

HS, YZ, and CM contributed to conception and design of the study. YZ organized the database. SS and YZ performed the statistical analysis and wrote the manuscript. All authors contributed to manuscript revision and read and approved the submitted version.

## Conflict of Interest

The authors declare that the research was conducted in the absence of any commercial or financial relationships that could be construed as a potential conflict of interest.
