# Supplementary material for: Universal and Specific Predictors of Chinese Children With Dyslexia – Exploring the Cognitive Deficits and Subtypes
Source: Front Psychol. 2020 Jan 8;10:2904. doi: 10.3389/fpsyg.2019.02904 (PMC6960230; doi:10.3389/fpsyg.2019.02904)
Supplement: Supplementary file 1 [file Table_1.DOCX]

Supplementary Material

# Supplementary Tables

Table S1 Simple correlations among cognitive and reading measures for control and dyslexic children

|  | PD | RAN | MP | CR |
| --- | --- | --- | --- | --- |
| PD | -- | 0.275*** | 0.410*** | 0.325*** |
| RAN | 0.126 | -- | 0.038 | 0.204** |
| MP | 0.231*** | 0.195** | -- | 0.196** |
| CR | 0.171** | 0.303*** | 0.374*** | -- |

*Note*: PD = Phoneme Deletion, RAN = Rapid Automatized Naming Digits, MP = Morphological Production, CR= Chinese Character reading.

Correlations for control children are presented below the diagonal, and correlations for dyslexic children are presented above the diagonal.

**, *p* < .01; ***, *p* < .001.

Table S2 The logistic regression model for predicting dyslexia by only Phoneme Deletion and RAN digits

|  | Estimate | S.E. | Odds Ratio (OR) | *p* value | 95% C.I. for OR | |
| --- | --- | --- | --- | --- | --- | --- |
|  |  |  |  |  | present study | Landerl et al., 2013 |
| PD | -0.95 | 0.11 | 0.39 | <.001 | [0.31, 0.48] | [0.31, 0.41] |
| RAN | -0.90 | 0.14 | 0.41 | <.001 | [0.31, 0.53] | [0.31, 0.41] |
